# Toxicity remission of PAEs on multireceptors after molecular modification through a 3D-QSAR pharmacophore model coupled with a gray interconnect degree method

**DOI:** 10.3906/kim-2008-38

**Published:** 2021-04-28

**Authors:** Xinyi CHEN, Yu LI

**Affiliations:** 1 MOE Key Laboratory of Resource and Environmental System Optimization, Ministry of Education,North China Electric Power University, Beijing China

**Keywords:** Gray interconnect degree, phthalate acid esters, multireceptor toxicity, pharmacophore model, molecule modification

## Abstract

In the proposed model, the gray interconnect degree method was employed to process the acute toxicity values of phthalate acid esters (PAEs) to green algae, daphnia, mysid, and fish (predicted by EPI Suite software) and to obtain the comprehensive characterization value of the multireceptor toxicity effect (MTE) of PAEs. The 3D-QSAR pharmacophore model indicated that hydrophobic groups significantly affected the MTE of PAEs. Based on this, 16 PAEs derivative molecules with significantly decreased comprehensive characterization value (more than 10%) of the toxic effects of multireceptors were designed. Among them, 13 PAEs derivative molecules reduced the toxicity values (predicted by the EPI Suite software) of four receptor organisms to varying degrees. Finally, two derivative molecules from PAEs were screened and could exist stably in the environment. The derivative molecule’s reduced toxicity to the receptor was obtained through molecular docking methods and simulated the PAEs’ primary metabolic response pathways. The above research results break through the pharmacophore model’s limitation of only being suitable for the single effect of pollutants. Its application provides a new theoretical verification basis for expanding the multieffect pharmacophore model.

## 1. Introduction

Phthalate acid esters (PAEs) are widely used organic substances. Their chemical structure consists of a planar aromatic hydrocarbon and two fatty side chains (4-15 carbon alkyl groups, C_n_H_2n+1_) [1]. In recent years, microplastic pollution has caused widespread concern, and additives in plastics, such as phthalates, bisphenol A, and poly brominated diphenyl ethers, also enter the water environment withplastics’ physical and chemical degradation which has toxic effects on aquatic life [2]. As a plasticizer, PAE molecules are used widely in hundreds of daily necessities, such as commodity packaging bags, cleaning solutions, adhesives, and soaps [3,4]. The total amount of PAEs consumed annually worldwide is as much as 1.5 × 10^11^ kg [5]. PAEs and the plastic matrix are not bonded in the form of covalent bonds but are connected by hydrogen bonds or van der Waals forces [6] which are easily released from products and migrate to both food and the environment, so they can be detected in the atmosphere [7], water [8], soil [9], and organisms [10]. PAEs are easily soluble in organic media but are difficult to dissolve in water, with a strong resistance to environmental degradation. In addition to acute and chronic toxicity to organisms, PAEs also cause “three effects” (carcinogenic, teratogenic, and mutagenic) [11,12]. The United States Environmental Protection Agency (EPA) listed DEHP, BBP, DBP, DEP, DOP, and DMP (Table 1) as priority toxic pollutants in 1977 [13]. China also suggested that DMP, DOP, and DBP should be included in the priority pollution control list [14].

**Table 1:** Full names and abbreviations of 23 PAEs molecules.

Abbreviations	Full name	Abbreviations	Full name
DEHP	Bis (2-ethylhexyl) phthalate	DEP	Diethyl phthalate
BBP	Benzyl butyl phthalate	DOP	Dinoctyl phthalate
DBP	Dibutyl phthalate	DMP	Dimethyl phthalate
DIBP	Diisobutyl phthalate	DIDP	Diisodecyl phthalate
BMPP	Bis (4-methyl-2-pentyl) phthalate	DHXP	Dihexyl phthalate
DAP	Diallyl phthalate	DIHXP	Diisohexyl phthalate
DMEP	Bis (2-methoxyethyl) phthalate	DPP	Dipentyl phthalate
DPrP	Dipropyl phthalate	DINP	Diisononyl phthalate
DIPP	Diisopentyl phthalate	DIPrP	Diisopropyl phthalate
DNP	Dinonyl phthalate	DIHP	Diheptyl phthalate
DUP	Diundecyl phthalate	DIOP	Di-isooctyl phthalate
DTDP	Ditridecyl phthalate		

During production and use, many PAE compounds enter the water environment through wastewater discharge, rainwater erosion, and atmospheric wet and dry settlement [15]; as a result, PAE concentrations in most rivers and lakes exceed 8.0 μg/L, over the limit of surface water environmental quality standards [16]. Due to PAE compounds’ low vapor pressure, their volatilization loss in the water environment is small. PAEs have strong adsorption and an affinity for aquatic organisms, which endangers aquatic organisms’ health [17]. With algae as the primary producer, toxic substances in the water environment use it as a medium to pass through the food chain to higher organisms [18]. Copepods play an important intermediary role in transmitting pollutants along the food chain [19]. Fish and crustaceans are the most dominant groups in swimming animal communities [20]. Studies have shown that PAEs can cause damage to algae’s organelles and antioxidant systems, resulting in cell deformities and inhibiting algae growth [21]. Long-term exposure to DEHP has a certain inhibitory effect on total reproductive mass, average reproductive mass, and population growth of the large salamander F3 generation [22]. PAEs also have harmful effects on the reproductive and endocrine systems of fish and crustaceans [23], and Patyna found that continuous exposure to low DBP concentrations seriously affect the fertility of Japanese sturgeon offspring [24]. The above literature mainly focuses on PAEs’ acute toxicity in a certain organism, and only focuses on some PAE molecules in terms of their exposure pathway, toxicity, and performance of a single biological receptor. The research on the toxicity’s molecular mechanism is insufficient.In view of PAEs’ increasingly widespread application from an environmental pollution control perspective, it is important to carry out multireceptor low-toxicity activity PAE molecule joint regulation. Therefore, algae, invertebrates, and fish must be included in toxicological data, this article selects four common aquatic organisms (green algae, daphnia, mysid, and fish) that represent different nutritional levels in the water environment to study PAEs’ comprehensive toxicity effects on four aquatic organisms and modify multireceptor low-toxicity PAE molecule.

QSAR, as a technology to quantitatively reveal compounds’ toxicity and biological activity, can use the validated pharmacophore model [25]. Song et al. [26] proved a pharmacophore model to study the acute toxicity of six naphthoquinone compounds to daphnia magna. The results showed that the compounds’ hydrophobicity had a great effect on receptor toxicity. Wang et al. [27] used hydrophobic groups to establish a pharmacophore model of the toxicity of perfluoro carboxylic acids to photobacterium, and the model regression coefficient was high. Qiu et al. [28] used a pharmacophore model to perform hydrophobic group substitution reactions on nine common PAE molecules and selected derivatives, with significantly enhanced Raman characteristic vibration spectra of PAEs. Jiang [29] proved the pharmacophore model could construct a POPs characteristic regulation scheme for PBDEs, and carried out modification designs of representative homologs, which confirmed the pharmacophore model’s feasibility in molecular modification. Therefore, in this paper, studying the regulation scheme of the MTE of PAEs to multireceptors can be based on above pharmacophore model design method. In view of the limitation of the pharmacophore model’s dependent variable as the pollutant’s single pollution effect, this paper uses a grey interconnect degree [30] to deal with the aquatic receptors’ toxicity values and calculate the toxicity comprehensive characterization values of PAEs to multireceptors. It is applied to the construction of the pharmacophore model of the MTE of PAEs and the modification design of multireceptor low-toxicity PAEs’ derivative molecules, which provide a theoretical basis for constructing a multireceptor comprehensive toxicity effect model of PAEs.

## 2. Materials and methods

### 2.1. Sources of data

The ECOSAR toxicity prediction module in EPI Suite software was used to predict the toxicity of 14 PAEs molecules to four organisms (green algae, daphnia, mysid, and fish),expressed as the concentration for a 50% maximal effect (EC50) or 50% lethal concentration (LC50), as shown in Table 2.

**Table 2:** Predicted acute toxicity values of 14 PAEs molecules to 4 recipient organisms.

PAEs	Green algae	Daphnid	Mysid	Fish
	96-EC50	48-LC50	96-LC50	96-LC50
	mg/L	mg/L	mg/L	mg/L
DEHP	0.00157	0.01	0.000419	0.01
DIDP	0.0000758	0.000669	0.0000115	0.000787
DNOP	0.00124	0.008	0.000317	0.008
DPP	0.111	0.463	0.067	0.327
DCHP	0.045	0.206	0.023	0.155
DUP	0.0000131	0.000138	0.00000143	0.000183
BCHP	0.149	0.602	0.095	0.417
BDP	0.006	0.032	0.0019	0.028
BMPP	0.032	0.15	0.015	0.116
BOP	0.025	0.121	0.011	0.095
DINP	0.000272	0.002	0.0000526	0.002
DIPP	0.141	0.573	0.088	0.398
DNDP	0.0000598	0.00054	0.0000087	0.000646
HEHP	0.006	0.035	0.002	0.03

### 2.2. Calculation of comprehensive characteristic values of the MTE of PAEs using the gray interconnect degree method

A gray relation analysis (GRA) is a multifactor statistical analysis method, based on the similarity or dissimilarity of development trends between factors, which is used to measure the degree of correlation between factors [31]. The GRA results were obtained by the correlation between an indicator and factors that affect the indicator because this method involves longitudinal averaging of gray interconnect coefficients [32]. However, the PAEs molecules (indicators)’ comprehensive toxicity effect on four aquatic organisms (factors) was studied in this paper. It was not necessary to obtain the order of the degree of influence between each factor and the indicator, which requires horizontal averaging of gray interconnect coefficients.

Because the dimensions of the acute toxicity prediction values of PAEs are the same, no dimensionless processing was required. The acute toxicity classification standard (LC50/EC50 < 1.0 mg/L) was used as the reference sequence, X_0_, and the toxicity values of four organisms to green algae, daphnia, mysid, and fish were used as the comparison sequence, X_i_ (i = 1,2,3,4), the weight of the four groups of comparison sequences was set to 25%. After obtaining the absolute difference between the corresponding points of the reference sequence, X_0_, and the comparison sequence, X_i_ (i = 1,2,3,4, k = 1,2,3, … ,14), and substituting each column’s maximum and minimum values of the absolute difference into Eq. (1) to calculate gray interconnect coefficients (ξ_0i_(k)) of the four comparison sequences (X_i_) and the reference sequence (X_0_), where ρ is the resolution coefficient, *ρ*∈(0,1), and generally takes a value of* ρ *= 0.5.

(1)ξ0i(k)=minimink|x0(k)-xi(k)|+ρmaximaxk|x0(k)-xi(k)||x0(k)-xi(k)|+ρmaximaxk|x0(k)-xi(k)|

Eq. (2) was used to calculate the average value of the gray interconnect coefficients horizontally to obtain the gray interconnect degree, y_ok_, of PAEs and four aquatic organisms. This was used as a comprehensive characterization of the MTE of PAEs, where n = 4.

(2)yok=1n∑i=1nξoi(k)

### 2.3. Construction method of the pharmacophore model of the multireceptor low-toxicity comprehensive effect of PAEs

The structural formulas of 14 PAE molecules were drawn by SYBYL-X2.0 software, entering the molecular construction mode from “sketch” in the toolbar, then optimizing the PAE molecules’ force field after drawing, selecting the molecules’ lowest energy conformation as the dominant stable conformation, optimizing each molecule’s energy in the “Tripos” force field with the molecular program “minimize” and selecting “Gasteiger–Huckel” from the “charges” option menu. Using Powell’s energy gradient method, “minimize details” was clicked, selecting the maximum number of repetitions (max. iterations) to 10,000, reducing the energy convergence limit (gradient) to 0.005 [33]. The “gradient” value is a termination criterion and, if the gradient difference calculates twice consecutively below this value, the calculation is terminated and the molecular structure optimization is completed.

The “3D-QSAR pharmacophore model generation” module in Discovery Studio 4.0 software was used to build the pharmacophore model [34]. The selected model’s pharmacophore characteristics include: hydrogen bond donor (HBD), hydrogen bond acceptor (HBA), hydrophobic group (H), hydrophobic ring (HA), and ring aromatic (RA). The parameters for generating all molecular conformations were set as follows: “conformation generation” selected the best mode (best), the default energy cutoff of “energy threshold” was 20 kcal/mol, the “maximum conformations” was 255, the “minimum interfeature distance” was 1.5, the number of pharmacophore features was 0–5, and the energy threshold of each homolog to generate a similar conformation was 10, while other parameters adopted default values.

The “Hypo Gen” module in Discovery Studio 4.0 software was selected to evaluate the constructed pharmacophore model. “Cost function”, one of the model’s evaluation indicators, was used to express and evaluate the model’s complexity and chemical characteristics as well as errors between each model’s predicted values and experimental data. Each pharmacophore model had its own total consumption (total cost). According to Occam’s Razor [35], the lower the “total cost” value, the closer it is to the “fixed cost” value, so the pharmacophore model is more reliable. “Configuration cost”, another important parameter, is determined by the model’s spatial complexity. The “configuration cost” value of a significant pharmacophore model should not be greater than 17 [36]. The larger the model correlation coefficient “R2” (> 0.7), the more predictive the pharmacophore model, and the more likely it is to meet the analytical needs [37]. In addition, “root mean square,” “fit value,” and “error” can be used as the pharmacophore model’s evaluation indices.

### 2.4. Molecular docking and quantum chemical calculation methods

Molecular docking supposes that the binding between the ligand and the receptor conforms to the “lock and key principle”, which satisfies the matching of spatial shape and energy, and finally obtains the optimal binding mode and stable composite conformation. Herein, the Lib–Dock quick docking method in Discovery Studio 4.0 softwarewas used [38]. The Poling algorithm performs a conformation search on the ligand molecule and then analyzes the binding site of the receptor and uses the grid-like algorithm to generate a series of polar and nonpolar hot spots. Finally the conformation and hot spots are matched with the energy and geometry to obtain the docking result. Considering that the crystalline water molecule at the binding site may affect the binding of ligand receptor, the water molecule at the protein binding site is eliminated when docking. “Find sites from receptor cavities” under the “Define” module determines the possible binding sites for ligand receptors, followed by selecting “user specified” in “Docking preferences”, setting the maximum saved conformation to “10”, and the rest of the parameters are the default values. The docking result is expressed using the “Lib–Dock score.” The magnitude of the value represents the strength of the binding ability. 

The quantum chemistry calculations herein are based on the Gaussian 09 software package, the computer operating system used is Linux, and the Gaussian calculation results are displayed using the Gauss view 5.0 program. The DFT method is used to calculate the reactants, products, and transition state (TS) of the primary metabolic reaction at the B3LYP/6-31G(d) basis set level, and the reaction energy barrier (ΔE) of the primary metabolic pathway of PAEs molecules is calculated using Eq. (3). The TS has only one imaginary frequency, and the reaction path is verified through the intrinsic reaction coordinate [39].

(3)ΔE=ETS-∑Ereactant

## 3. Results and discussion

### 3.1. Calculation of comprehensive characterization of the MTE of PAEs

Using the gray interconnect degree to process the original data (predicted by EPI Suite software), the absolute difference between the corresponding points of X_0_ and X_i_, should be found, and the minimum and maximum values of each column’s absolute difference should be obtained (Table 3, for calculation validity, retaining six significant digits).

**Table 3:** Absolute difference between X_0_(k) and Xi(k).

k	|X_0_(k)-X1(k)|	|X_0_(k)-X2(k)|	|X_0_(k)-X3(k)|	|X_0_(k)-X4(k)|
1	0.990000	0.998430	0.990000	0.999581
2	0.999213	0.999924	0.999331	0.999989
3	0.992000	0.998760	0.992000	0.999683
4	0.673000	0.889000	0.537000	0.933000
5	0.845000	0.955000	0.794000	0.977000
6	0.999817	0.999987	0.999862	0.999999
7	0.583000	0.851000	0.398000	0.905000
8	0.972000	0.994000	0.968000	0.998100
9	0.884000	0.968000	0.850000	0.985000
10	0.905000	0.975000	0.879000	0.989000
11	0.998000	0.999728	0.998000	0.999947
12	0.602000	0.859000	0.427000	0.912000
13	0.999354	0.999940	0.999460	0.999991
14	0.970000	0.994000	0.965000	0.998000
Min(i)(k)	0.583000	0.851000	0.398000	0.905000
Max(i)(k)	0.999817	0.999987	0.999862	0.999999

We substitute into Eq. (1) to calculate the gray interconnect coefficient ξ_0i_(k) of each corresponding point and then obtain the gray interconnect degree y_0k_ of PAEs and four aquatic organisms from Eq. (2) (Table 4).

**Table 4:** Grey interconnect coefficients of PAEs to 4 receptor organism.

ξ01(k)	ξ02(k)	ξ03(k)	ξ04(k)	y_0k_
0.7268	0.9016	0.6027	0.9369	0.7920
0.7224	0.9007	0.5989	0.9367	0.7897
0.7259	0.9014	0.6019	0.9369	0.7915
0.9233	0.9726	0.8660	0.9805	0.9356
0.8052	0.9285	0.6940	0.9513	0.8447
0.7221	0.9007	0.5987	0.9367	0.7895
1.0000	1.0000	1.0000	1.0000	1.0000
0.7357	0.9043	0.6117	0.9379	0.7974
0.7825	0.9203	0.6652	0.9461	0.8285
0.7708	0.9159	0.6512	0.9436	0.8204
0.7229	0.9008	0.5994	0.9367	0.7900
0.9828	0.9941	0.9687	0.9950	0.9852
0.7223	0.9007	0.5989	0.9367	0.7896
0.7367	0.9043	0.6130	0.9379	0.7980

Comprehensive characterization values of the MTE of PAEs are seen in Table 5.

**Table 5:** Comprehensive characterization values of 14 PAEs’ MTE.

PAEs	DEHP	DIDP	DNOP	DPP	DCHP	DUP	BCHP
Value	0.7920	0.7897	0.7915	0.9356	0.8447	0.7895	1.0000
PAEs	BDP	BMPP	BOP	DINP	DIPP	DNDP	HEHP
Value	0.7974	0.8285	0.8204	0.7900	0.9852	0.7896	0.7980

### 3.2. Construction and evaluation of pharmacophore model of the multireceptor low-toxicity comprehensive effect of PAEs

Herein, 14 PAE molecules were divided into 10 training set molecules used for the construction of pharmacophore models, and four test set molecules were used to validate the pharmacophore models.The pharmacophore models with good performance parameters are listed in Table 6.

**Table 6:** Five pharmacophore models statistical data constructed by Hypo Gen.

Hypo NO.	Total cost	RMS	Correlation	Feature
1	51.610	0.055	0.85	HBA, H, HA
2	51.611	0.056	0.67	HBA*2, RA
3	51.612	0.057	0.58	HBA*2, H
4	51.612	0.058	0.56	HBA*2, RA
5	51.613	0.059	0.60	HBA*2, RA
Fixed cost	33.636	Configuration	16.834	

HBA: hydrogen bond acceptor; H: hydrophobic;

It was demonstrated that Hypo 1 had the best evaluation score among the five models. “Total cost” value (51.61) and “RMS” value (0.055) were the smallest, “total cost” was closest to “fixed cost” (33.636), “configuration” value was 16.834 (<17) and “correlation” (0.85) was closest to 1, the absolute value of “error” value of the molecules was less than 2 [40], and was within the tolerance range. Therefore, the model is significant and meets the requirements, so Hypo 1 was selected as the optimal pharmacophore model. The model has a hydrogen bond acceptor, a hydrophobic group, and a hydrophobic ring. The test set was used to verify this pharmacophore model, the “fit values” of the PAEs molecules were high, and the absolute “error” values were less than 2 (shown in Table 7), which shows that Hypo 1 had a stable prediction ability for PAE molecules other than the training set.

**Table 7:** Comprehensive evaluation values of Hypo 1 and PAEs’ training set, test set.

	PAEs	Fit value	Estimated	Active	Error
Training set	DINP	5.92	0.72	0.79	–1.10
	DEHP	5.91	0.74	0.792	–1.06
	BDP	5.88	0.79	0.797	–1.01
	BOP	5.87	0.80	0.82	–1.03
	HEHP	5.87	0.81	0.798	1.01
	DNOP	5.86	0.82	0.792	1.03
	DPP	5.85	0.84	0.936	–1.11
	BMPP	5.84	0.87	0.829	1.05
	DCHP	5.82	0.90	0.845	1.07
	BCHP	5.74	1.09	1.0	1.09
Test set	DIDP	5.96	0.66	0.79	–1.20
	DUP	5.85	0.84	0.79	1.06
	DNDP	5.82	0.90	0.79	1.13
	DIPP	5.77	1.02	0.985	1.03

### 3.3. Determination of substitution groups and substitution sites of target molecules, DINP, and DEHP, based on the Hypo 1 optimal pharmacophore model

**Figure 1:**
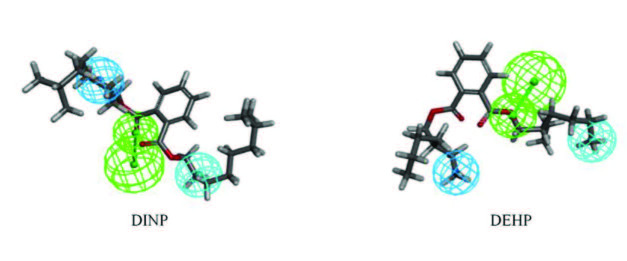
Hypo 1 pharmacophore model overlay with target molecules –DINP, DEHP.

DEHP, which has priority control of pollutants, and DINP, which has the largest comprehensive characterization of the MTE of PAEs in the training set, were selected as target molecules to determine the molecular modification site. Derivative molecules were designed based on this. Figure 1 shows the superposition relationship of Hypo 1 with DINP and DEHP. The Hypo 1 pharmacophore model contained one hydrogen bond acceptor (green), one hydrophobic (light blue), and one hydrophobicity ring (dark blue); their positions on the molecular structure can be seen. 

**Figure 2:**
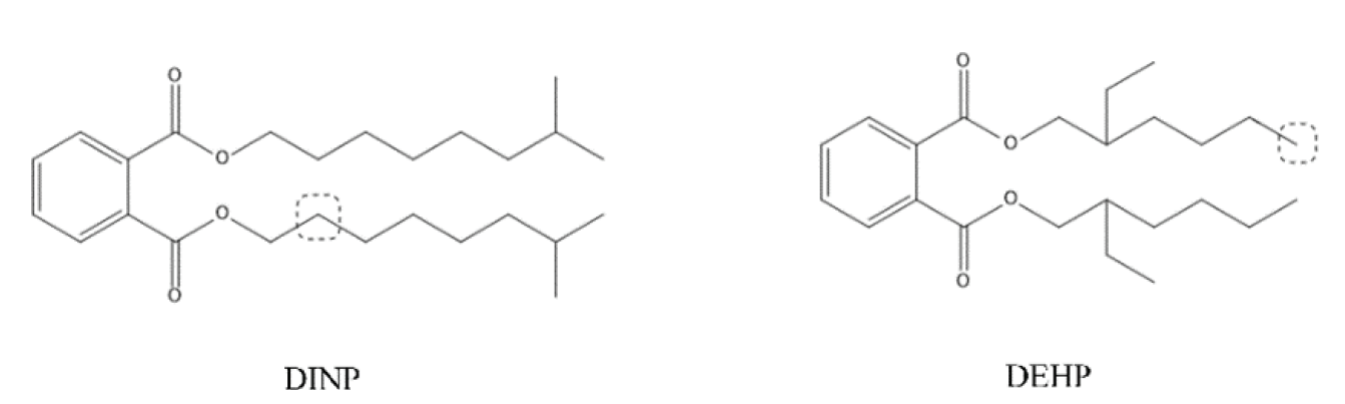
Location of hydrophobic substitution sites for target molecules DINP and DEHP.

Among them, the hydrophobic group was at the position of carbon atom No.2 of the branch chain, connected to the No.2 carboxyl oxygen atom of DINP molecule, the position of carbon atom No.6 of the branch chain connected to the No.1 carboxyl oxygen atom of the DEHP molecule (shown in Figure 2). Therefore, introducing a hydrophobic group at branch positions can affect the PAEs’ toxic activity. The positions of the substitution groups, introduced by DINP and DEHP, are shown in Figure 2; that is, molecular modification of this site was determined, which provides a basis for further screening derivative molecules for the MTE of PAEs.

### 3.4. Molecular modification of PAE derivatives based on the multireceptor low-toxicity pharmacophore model

Eleven common hydrophobic group were selected as substituent groups: methyl (–CH_3_), ethyl (–CH_2_CH_3_), propyl (–CH_2_CH_2_CH_3_), vinyl (–CH=CH_2_), phenyl (–C_6_H_5_), methoxyl (–OCH_3_), hypochlorite (–Cl), fluoride (–F), bromo (–Br), sulfydryl (–SH), and nitro (–NO_2_), to monosubstituted modification for DINP and DEHP, obtaining 22 modified derivative molecules. The constructed optimal pharmacophore model, Hypo 1, was used to predict the comprehensive characterization value of the MTE of PAE derivative molecules and compared with the toxicity comprehensive characterization values of corresponding target molecules,as shown in Table 8. The results showed that 16 PAE derivative molecules with toxicity comprehensive characterization values increased by more than 10%, including nine DINP derivative molecules (an increase of 11.95%–208.12%), and seven DEHP derivative molecules (an increase of 13.02%–48.07%), indicating that the toxicity of 16 derivative molecules was significantly lower than the target molecule.

**Table 8:** Prediction of comprehensive characterization values of PAEs derivatives’ MTE.

Compounds	Estimated	Change rate	Compounds	Estimated	Change rate
DINP	0.7171		DEHP	0.7441	
DINP-CH_3_	0.8214	14.54%	DEHP-CH_3_	0.7442	0.01%
DINP-CH_2_CH_3_	0.6997	–2.43%	DEHP-CH_2_CH_3_	1.0099	35.72%
DINP-CH_2_CH_2_CH_3_	0.8028	11.95%	DEHP-CH_2_CH_2_CH_3_	0.7456	0.20%
DINP-CH=CH_2_	0.8247	15.00%	DEHP-CH=CH_2_	0.8410	13.02%
DINP-C_6_H_5_	0.8124	13.29%	DEHP-C_6_H_5_	0.8641	16.13%
DINP-OCH_3_	1.1392	58.86%	DEHP-OCH_3_	1.1018	48.07%
DINP-CI	0.6761	–5.72%	DEHP-CI	0.7906	6.25%
DINP-F	1.0018	39.70%	DEHP-F	0.9070	21.89%
DINP-Br	0.9851	37.37%	DEHP-Br	0.8554	14.96%
DINP-SH	2.2095	208.12%	DEHP-SH	0.7712	3.64%
DINP-NO_2_	1.6933	136.13%	DEHP-NO_2_	0.8929	20.00%

### 3.5. Evaluation and verification of multireceptor comprehensive toxicity of PAE derivatives

#### 3.5.1. Evaluation and verification of the MTE of PAE derivatives based on the EPI database

The ECOSAR module in the EPI Suite software was used to predict the above 16 PAE derivative molecules’ toxicity values to multireceptor model (green algae, daphnia, mysid, and fish), taking the negative logarithmic values, as shown in Table 9. The DINP derivative molecules’ predicted toxicity to the multireceptors was lower than that of the target molecule (decreased by: green algae, 43.91%–93.45%; daphnia, 53.03%–111.83%; mysid, 43.96%–92.12%; and fish, 50.10%–104.63%). DEHP-OCH_3_, DEHP-F, DEHP-Br, and DEHP-NO_2_ in the DEHP derivative molecules had lower toxicity prediction values for the multireceptor model than the target molecule (decreased by: green algae, 3.75%–34.96%; daphnia, 8.88%–44.88%; mysid, 5.57%–34.22%; and fish, 7.31%–40.65%), and the decline of multireceptors was close to 1:1:1:1. Therefore, a total of 13 PAE derivative molecules were screened, with a significant reduction in toxic activity.

**Table 9:** Negative logarithmic predicted values of PAEs derivative molecules on green algae, daphnia, mysid, and fish based on the EPI database.

Compounds	Green algae	Change rate	Daphnid	Change rate
	EC50 (mg/L)		LC50 (mg/L)	
DINP	3.5654		2.6990	
DINP-CH_3_	1.2218	–65.73%	0.5784	–78.57%
DINP-CH_2_CH_2_CH_3_	1.8861	–47.10%	1.1612	–56.98%
DINP-CH=CH_2_	1.4559	–59.16%	0.7852	–70.91%
DINP-C_6_H_5_	2.0000	–43.91%	1.2676	–53.03%
DINP-OCH_3_	0.3251	–90.88%	–0.2350	–108.71%
DINP-F	0.8356	–76.56%	0.2262	–91.62%
DINP-Br	1.0410	–70.80%	0.4056	–84.97%
DINP-SH	0.8182	–77.05%	0.2097	–92.23%
DINP-NO_2_	0.2336	–93.45%	–0.3193	–111.83%
DEHP	2.8041		2.0000	
DEHP-CH_2_CH_3_	3.1331	11.73%	2.3010	15.05%
DEHP-CH=CH_2_	3.0400	8.41%	2.2218	11.09%
DEHP-C_6_H_5_	3.5918	28.09%	2.6990	34.95%
DEHP-OCH_3_	1.9208	–31.50%	1.1871	–40.65%
DEHP-F	2.3979	–14.48%	1.6383	–18.09%
DEHP-Br	2.6990	–3.75%	1.8239	–8.80%
DEHP-NO_2_	1.8239	–34.96%	1.1024	–44.88%
Compounds	Mysid	Change rate	Fish	Change rate
	LC50 (mg/L)		LC50 (mg/L)	
DINP	4.2790		2.6990	
DINP-CH_3_	1.4949	–65.07%	0.7100	–73.70%
DINP-CH_2_CH_2_CH_3_	2.3010	–46.23%	1.2441	–53.90%
DINP-CH=CH_2_	1.7696	–58.65%	0.8996	–66.67%
DINP-C_6_H_5_	2.3979	–43.96%	1.3468	–50.10%
DINP-OCH_3_	0.4425	–89.66%	–0.0453	–101.68%
DINP-F	1.0410	–75.67%	0.3830	–85.81%
DINP-Br	1.3010	–69.59%	0.5436	–79.86%
DINP-SH	1.0223	–76.11%	0.3665	–86.42%
DINP-NO_2_	0.3372	–92.12%	–0.1248	–104.63%
DEHP	3.3778		2.0000	
DEHP-CH_2_CH_3_	3.7670	11.52%	2.3010	15.05%
DEHP-CH=CH_2_	3.6576	8.28%	2.2218	11.09%
DEHP-C_6_H_5_	4.3170	27.80%	2.6990	34.95%
DEHP-OCH_3_	2.3010	–31.88%	1.2676	–36.62%
DEHP-F	2.9208	–13.53%	1.6990	–15.05%
DEHP-Br	3.1898	–5.57%	1.8539	–7.31%
DEHP-NO_2_	2.2218	–34.22%	1.1871	–40.65%

#### 3.5.2. Evaluation and verification of the MTE of PAE derivativesbased on the single receptor pharmacophore model

Based on the negative logarithm of toxicity values (predicted by EPI Suite software) of 14 PAEs on multireceptors as data sources, the abovementioned PAEs’ toxicity comprehensive effect pharmacophore model construction method was used to construct green algae, daphnia, mysid, and fish’ single receptor optimal pharmacophore models, as shown in Table 10. The “configuration” values of the four pharmacophore models were 16.674 (<17), while the “correlation” values were all greater than 0.7. This shows that the established pharmacophore model exhibits stable prediction ability.

**Table 10:** Construction results of toxicity activity pharmacophore model of PAEs on green algae, daphnia, mysid, and fish.

Hypo No.	Configuration	Total cost	RMS	Correlation	Features
Hypo for green algae	16.674	52.043	0.348	0.72	HBA*2, H
Hypo for daphnid	16.674	52.270	0.409	0.87	HBA*2, H, HA
Hypo for mysid	16.674	51.738	0.246	0.82	HBA*2, H*2
Hypo for fish	16.674	51.787	0.265	0.90	HBA*2, H, RA

The above PAEs single receptor pharmacophore model was used to predict the PAEs derivative molecules’ toxic activity on the corresponding receptors (negative logarithmic values, Table 11). Among these, DINP-C_6_H_5_ and DEHP-F derivatives showed a consistent decrease in toxic activity on the multireceptor model, and this was consistent with the trend in the predicted value of the comprehensive effect pharmacophore model, which further verifies the reliability of the PAEs’ multireceptor toxicity comprehensive effect pharmacophore model.

**Table 11:** Toxicity prediction value of PAEs derivative molecules on green algae, daphnia, mysid, and fish based on pharmacophore model.

Compounds	Green algae	Change rate	Daphnid	Change rate
DINP	2.3039		1.3371	
DINP-CH_3_	2.0049	–12.98%	2.0082	50.19%
DINP-CH_2_CH_2_CH_3_	2.1938	–4.78%	2.9862	123.33%
DINP-CH=CH_2_	1.2773	–44.56%	2.4678	84.56%
DINP-C_6_H_5_	2.1943	–4.76%	0.394	–70.53%
DINP-OCH_3_	2.1396	–7.13%	1.9394	45.05%
DINP-F	1.4256	–38.12%	2.3887	78.65%
DINP-Br	1.3281	–42.35%	1.100	–17.73%
DINP-SH	1.6563	–28.11%	5.5234	313.09%
DINP-NO_2_	1.7235	–25.19%	1.1611	–13.16%
DEHP	2.4583		2.5771	
DEHP-OCH_3_	2.0141	–18.07%	5.3772	108.65%
DEHP-F	2.3623	–3.91%	2.2673	–12.02%
DEHP-Br	1.7537	–28.66%	1.9605	–23.93%
DEHP-NO_2_	1.9213	–21.85%	0.7316	–71.61%
Compounds	Mysid	Change rate	Fish	Change rate
DINP	3.0064		1.8487	
DINP-CH_3_	1.9758	–34.28%	2.3857	29.05%
DINP-CH_2_CH_2_CH_3_	2.4324	–19.09%	1.3578	–26.55%
DINP-CH=CH_2_	2.9583	–1.60%	1.1800	–36.17%
DINP-C_6_H_5_	2.0074	–33.23%	0.7200	–61.05%
DINP-OCH_3_	2.2089	–26.52%	3.1838	72.23%
DINP-F	1.9745	–34.32%	3.1892	72.52%
DINP-Br	2.4098	–19.84%	2.4892	34.65%
DINP-SH	1.9857	–33.95%	1.5304	–17.21%
DINP-NO_2_	2.1128	–29.72%	2.1955	18.77%
DEHP	2.5511		1.2037	
DEHP-OCH_3_	1.9799	–22.39%	1.2720	5.67%
DEHP-F	2.2677	–11.11%	0.7456	–38.06%
DEHP-Br	5.0185	96.73%	0.9164	–23.87%
DEHP-NO_2_	2.7852	9.18%	0.4750	–60.54%

### 3.6. Evaluation of functional properties andpersistent organic pollutants (POPs) properties of PAE derivatives 

#### 3.6.1. Evaluation of functional properties of PAE derivatives 

The functional characteristics of PAE molecules include stability and insulation. The “total energy,” “energy gap” (which is the difference between the highest occupied orbital energy–E_HOMO_, and the lowest empty orbital energy–E_LUMO_ of the molecule [41]), “frequency” of the target molecule, and its derivative molecules were calculated using Gaussian software. The “total energy” value represents stability, the “energy gap” value represents insulation, and the larger the energy gap value, the stronger the insulation. At the same time, the “frequency” value (>0) was used to evaluate whether the derivative molecules can exist stably in the environment [42]. As can be seen from Table 12, in the designed PAEs derivative molecules, the “total energy” value of DINP-C_6_H_5_ decreased 6.34%, while the DEHP-F increased, but the increase was small (<5%), indicating the PAEs derivative molecules’ stability had improved or remained unchanged compared with the target molecule. The “energy gap” value demonstrated a smaller change, indicating that the PAEs derivative molecules’ stability had increased, while insulation was less affected. Both PAEs derivatives had “frequency” values greater than zero, indicating that their structures could exist stably in the environment.

**Table 12:** Evaluation of stability and insulation properties of PAEs derivatives.

	Compounds	Stability		Insulation		Frequency (cm–1)
		Total eenergy(a.u.)	Change rate(%)	Energy gap(eV)	Change rate(%)	
Before modification	DINP	–1317.02		5.51		7.60
	DEHP	–1238.39		5.56		11.39
After modification	DINP-C_6_H_5_	–1233.51	–6.34	5.18	–5.99	17.73
	DEHP-F	–1298.30	4.84	5.49	–1.26	5.44

#### 3.6.2. Evaluation of persistent organic pollutants (POPs) properties of PAE derivatives

EPI Suite software was used to predict the bioaccumulation, long distance migration, and persistence of two PAE derivative molecules [43]. Based on POPs characteristic parameter values of DINP and DEHP, from the analysis in Table 13, the “LOGK_OW_” values of the two PAE derivative molecules had decreased by 6.56%–22.84%. The “LOGK_OA_” values had also decreased by 8.88%–12.56%, indicating that PAE derivative molecules had significant bioaccumulation and long distance migration in the environment. Because the PAE molecule itself is not a persistent organic pollutant (half-life in air>two days), the increase in the “half-life” value of the PAE derivative molecules had no significant effect on its persistence in the environment.

**Table 13:** POPs characteristic parameter values of PAEs target molecules and derivative molecules.

	Compounds	Mobility		Bioaccumulation		Persistence	
		log KOA	Change rate (%)	log KOW	Change rate (%)	Half-life(hr)	Change rate (%)
Before modification	DINP	13.585		9.37		11	
	DEHP	12.557		8.39		11.7	
After modification	DINP-C_6_H_5_	12.378	–8.88	7.23	–22.84	14.2	29.1
	DEHP-F	10.980	–12.56	7.84	–6.56	12.8	9.4

### 3.7. Analysis of the mechanism of PAE derivatives with low multireceptor toxicity

#### 3.7.1. Analysis of toxicity mechanism of PAE derivatives based on molecular docking

Under external stress, a large number of oxygen radicals were generated in the algae cells. At this time, the antioxidant system was activated, and the peroxidase catalysis promptly removed a large amount of reactive oxygen species. PAEs caused oxidative damage to algae cells by acting on mitochondria (Mn-SOD) and cytoplasm (Cu/Zn-SOD) [44]. Glutathione (GSH) is an important antioxidant in living organisms. Copepods are rich in unsaturated fatty acids and, when environmental stress exceeds the capacity of copepods, unsaturated fatty acids are degraded and lipid peroxidation occurs [45]. Chitinase is closely related to shrimp growth, food digestion, and disease defense [46], while PAEs may have toxic effects on shrimp by affecting chitinase gene expression [47]. Peroxisome proliferator-activated receptors (PPARs) control many intracellular metabolic processes. Among these, PPAR-α receptors are abundantly expressed in liver cells, and activation is a necessary condition for phthalate compounds to cause toxic hepatic reactionsin fish [48]. This article downloaded the MN-SOD enzyme crystal structure (1BA9), glutathione peroxidase crystal structure (3DWV), chitinase crystal structure (3ZXX), and PPAR-α protein crystal structure (3KDT) from the PDB protein database, which respectively represent green algae, daphnia, mysid, and fish receptors, docked with PAE molecules before and after modification, and expressed the recipient organism’s toxic activity by the molecular docking ability [49].

The target molecules (DEHP, DINP) and derivative molecules (DEHP-F, DINP-C_6_H_5_) were molecularly docked with the four enzyme proteins by using Discovery Studio 4.0 software and the corresponding scoring function values were calculated. The lower the scoring function value, the weaker the binding ability between molecules and enzyme protein, and the lower the toxic effect on the recipient organism. Table 14 shows that the scoring function values of the two PAE derivative molecules docking with four enzyme proteins were lower than the target molecules (a decrease of 3.9%–19.8%), indicating that the designed PAE derivative molecules had a weaker receptor binding ability, reducing toxicity to the receptor organism.

**Table 14:** Scoring function values for the docking of PAEs target and derivative molecules with enzyme protein molecules.

	Compounds	1BA9	Changerate	3DWV	Changerate	3ZXX	Changerate	3KDT	Changerate
Beforemodification	DINP	97.701		66.242		74.518		142.43	
	DEHP	72.054		56.532		63.789		82.86	
After modification	DINP-C_6_H_5_	83.904	–14.1%	59.264	–10.5%	68.658	–7.9%	114.19	–19.8%
	DEHP-F	58.184	–19.2%	53.211	–5.9%	58.610	–8.1%	79.66	–3.9%

After the molecule binds to the receptor protein, it falls into the pocket formed by the amino acid residues around the receptor protein and reacts with the receptor mainly through hydrogen bonding, charge, or polar interaction, followed by the main chain and side chain of amino acids, generally interact with acceptor molecules in the form of hydrogen bonds. The docking results show that the main forces when PAEs and their derivatives bind to 3KDT and 1BA9 proteins are electrostatic force and van der Waals force, and the main forces, when they bind to 3DWV and 3ZZX proteins, include the electrostatic force, van der Waals force, and hydrophobic interaction. Compared with the target molecule, when the derivative molecule binds to the receptor protein, the number of surrounding amino acid residues that interact with it decreases and the binding ability becomes weaker. Therefore, it is possible to explain the decrease in the scoring function values for the binding of the derivative molecule to the receptor protein. When DINP-C_6_H_5_ binds to 3DWV, the number of surrounding amino acid residues that interact with 3DWV is larger than that of the target molecule; however, the scoring function value is lower. The possible cause for this is that when the target molecule is combined with 3DWV, it forms hydrogen bonds with amino acid residues TRPB137 and HOHB3105 and forms a π-bond interaction with TRPB137, whereas DINP-C_6_H_5_ only forms a π-bond interaction with TRPB137 when combined with 3DWV. 

#### 3.7.2. Analysis of toxicity mechanism of PAE derivatives based on metabolic response

**Figure 3:**
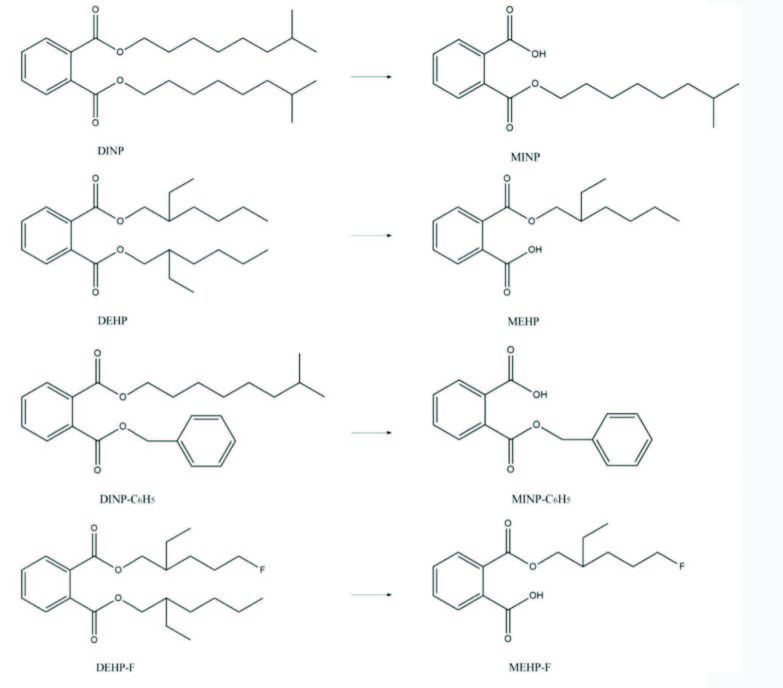
Simulation of primary metabolic pathways of PAEs target and derivative molecules in aquatic organisms.

The primary metabolite of PAEs, phthalate monoesters, has been detected in aquatic environments [50]. Ge Jian et al. [51] studied the metabolism of DEP, DBP, BBP, and DEHP in grass carp organs, and results showed that the main metabolites were corresponding phthalate monoesters. When studying the degradation products of black algae, Chen Bo [52] found that the phthalate monoesters, MBP and MEHP, were distributed in black algae. The primary metabolic pathway of PAEs in aquatic organisms is hydrolysis to the corresponding monoester compounds under the action of enzymes. According to these mimics the primary metabolic processes of PAEs, and their derivative molecules, in aquatic organisms,the products of DINP, DEHP, and derivative molecules DINP-C_6_H_5_, DEHP-F after primary metabolism are MINP, MEHP, MINP-C_6_H_5_, and MEHP-F (Figure 3).

Toxicity values of the primary metabolites of PAE target and derivative molecules (phthalate monoesters) to multireceptors were predicted using the EPI database (Table 15). The toxicity of PAE derivative monoester molecules was significantly lower than that of the target monoester molecules (green algae, 146.1%–2683.4%; daphnia, 125.5%–1880.3%; mysid, 188.4%–5070.1%; and fish, 112.6%–1473.4%).

**Table 15:** Toxicity prediction of PAEs target and derivative monoester molecules to green Algae, daphnia, mysid, and fish based on EPI database.

	Compounds	Green algaeEC50 (mg/L)	Change rate	DaphnidLC50 (mg/L)	Change rate
Beforemodification	MINP	1.929		7.553	
	MEHP	4.05		14.669	
Aftermodification	MINP-C_6_H_5_	53.691	2683.4%	149.574	1880.3%
	MEHP-F	9.966	146.1%	33.081	125.5%
	Compounds	MysidLC50 (mg/L)	Change rate	FishLC50 (mg/L)	Change rate
Beforemodification	MINP	1.288		5.131	
	MEHP	3.115		9.454	
Aftermodification	MINP-C_6_H_5_	66.591	5070.1%	80.731	1473.4%
	MEHP-F	8.984	188.41%	20.10	112.6%

Gaussian software was used to calculate the reaction energy barriers of the primary metabolic pathways of PAE target and derivative molecules and to determine whether the reaction could proceed, and how easy it was, by comparing the activation energy barriers in the transition states of the primary metabolic pathway response before and after molecular modification [53] as shown in Table 16. The reaction energy barrier of DINP was 51.77 KJ/MOL and DINP-C_6_H_5_ was 5.96 KJ/MOL; a reduction of 88.5% compared with DINP. The reaction energy barrier of DEHP was 4.08 KJ/MOL and DEHP-F was 0.31 KJ/MOL; a reduction of 92.4% compared with DEHP, indicating that the energy required for the first-order metabolism of PAE derivative molecules was greatly reduced compared with the target molecules, and the derivative molecules were more easily metabolized in aquatic organisms, causing the toxic activity of the organism to be reduced significantly.

**Table:** Energy barrier values of primary metabolic reactions of PAEs target molecules and derivative molecules.

	Compounds	Energy barrier(KJ/mol)	Changerate	Compounds	Energy barrier(KJ/mol)	Changerate
Before modification	DINP	51.77		DEHP	4.08	
After modification	DINP-C_6_H_5_	5.96	–88.5%	DEHP-F	0.31	–92.4%

## 4. Conclusion

Herein, the gray interconnect degree method assisted the PAE multireceptor low-toxicity effect and the pharmacophore model were established, passing validation of the traditional pharmacophore model and successfully applying it to the PAE multireceptor low-toxicity comprehensive effect of molecular modification. Based on the evaluation of the functional characteristics and POPs characteristics, two PAE derivative molecules were screened, with a reduction in comprehensive toxicity of 13.29% and 21.89%. Molecular docking and simulation methods of primary metabolic mechanisms in aquatic organisms confirmed the reason for the decrease in the multireceptor low-toxicity comprehensive effect of PAE derivative molecules. The method established in this article broke through the limitations of traditional pharmacophore models for single effect modeling of pollutants and provided theoretical support for building pharmacophore models that can simultaneously control multiple effects of pollutants.
